# Changes in Severity of Influenza A(H1N1)pdm09 Infection from Pandemic to First Postpandemic Season, Germany

**DOI:** 10.3201/eid1905.130034

**Published:** 2013-05

**Authors:** Nicola Lehners, Steffen Geis, Christoph Eisenbach, Kai Neben, Paul Schnitzler

**Affiliations:** Author affiliation: University of Heidelberg, Heidelberg, Germany

**Keywords:** influenza, influenza A(H1N1)pdm09 virus, risk factor, severe disease, age groups, viruses, expedited

## Abstract

We studied risk factors for a severe clinical outcome in hospitalized patients with laboratory-confirmed influenza A(H1N1)pdm09 infection at the University Hospital Heidelberg in the pandemic and first postpandemic seasons. We identified 102 patients in 2009–10 and 76 in 2010–11. The proportion of severely diseased patients dramatically increased from 14% in 2009–10 to 46% in 2010–11 as did the mortality rate (5%–12%). Patients in the first postpandemic season were significantly older (38 vs. 18 years) and more frequently had underlying medical conditions (75% vs. 51%). Overall, 50 patients (28%) had a severe clinical outcome, resulting in 14 deaths. Multivariate analysis showed that older male patients with chronic lung disease were at increased risk for a severe clinical outcome. In summary, the proportion of patients with severe disease and fatal cases increased in the postpandemic season. Therefore, patients with suspected infections should be promptly identified and receive early treatment.

Influenza pandemics have been associated with increased illness and death. Each pandemic is different, and areas of uncertainty always exist when an influenza virus emerges and becomes pandemic. In April 2009, the novel influenza A(H1N1)pdm09 virus emerged in Mexico and then spread rapidly throughout the world (*1*). Influenza is generally a self-limiting infection with systemic and respiratory symptoms that usually resolve after 3–6 days. Most persons infected with the 2009 influenza A(H1N1)pdm09 virus experienced uncomplicated illness with full recovery within 1 week, even without medical treatment; severe progressive disease developed in only a small subset of patients (*2*). Primary viral pneumonia was the most common finding in severe cases, but secondary bacterial infections played a role in ≈30% of fatal cases (*3*). Hospitalized patients were often affected by other medical conditions, such as diabetes and cardiovascular, neurologic and pulmonary diseases (*4*). Advances in therapy for malignancies, autoimmune disorders, and end-stage organ diseases have led to improved survival, but also to an increase in the number of immunosuppressed patients. These patients are particularly at risk for opportunistic and community-acquired infections, such as respiratory virus infections, resulting in considerable illness and death (*5*).

Although patients hospitalized with pandemic influenza A(H1N1)pdm09 infection had substantial severe illness, the overall number of deaths was lower than reported in the earliest studies. The overall number of deaths caused by influenza A(H1N1)pdm09 infection was similar to that caused by seasonal influenza and lower than that of previous pandemics (*6*). The most common cause of death was respiratory failure (*7*). Other reported causes of death included pneumonia, high fever leading to neurologic sequelae, dehydration from excessive vomiting and diarrhea, and electrolyte imbalance. Severe cases were most frequent in middle-aged patients, who often had coexisting conditions (*7*). Although to date there seems to be no major difference between the virulence of influenza A(H1N1)pdm09 strains and seasonal influenza (*8*) strains, a more aggressive course in specific populations, such as in young patients and pregnant women, has been reported (*9**,**10*). Further risk factors include obesity, chronic lung disease, chronic heart disease, chronic renal disease, diabetes mellitus, and severe immunosuppression (*4*,*11*,*12*). Contradictory findings have been reported in regard to varying disease severity during the pandemic season. Although some researchers did not observe any differences in disease severity between the first and second pandemic outbreaks in 2009 (*13*,*14*), another study showed a 4-fold increase in hospitalization and a 5-fold increase in number of deaths in the second wave (*15*). However, disease severity of postpandemic seasons has been rarely analyzed.

We performed a retrospective analysis of all patients with laboratory-confirmed influenza A(H1N1)pdm09 virus infection who were hospitalized at the University Hospital Heidelberg, Germany, in the pandemic season 2009–10 and the first postpandemic season 2010–11 to compare the rates of severely diseased patients in both seasons and to identify possible risk factors associated with severe clinical outcome.

## Methods

### Patients and Study Site

We conducted a retrospective cohort study of all patients admitted to the University Hospital Heidelberg, Germany, with laboratory-confirmed influenza A(H1N1)pdm09 infection from May 2009 through April 2011. We defined a case-patient as a hospitalized person with influenza-like illness and influenza A(H1N1)pdm09 virus infection confirmed by real-time PCR. We extracted positive influenza results from our laboratory information system and reviewed the patient charts for clinical and laboratory characteristics as described below. Additionally, we differentiated between pandemic and postpandemic season and severe and nonsevere infections. Severe disease was defined as either admission to the intensive care unit (ICU) or in-hospital death. Microbiologic studies, hospital and ICU admission criteria, and treatment decisions were not standardized but made at the discretion of the attending physicians.

### Data Collection

We collected data on demographic characteristics, coexisting conditions, clinical signs and symptoms, biochemical analyses, chest radiograph findings, antiviral and antibacterial therapy, concomitant and secondary bacterial infections as well as outcome, including death. Pneumonia was defined as the presence of a new infiltrate shown on a chest radiograph plus fever (temperature >38°C) and respiratory symptoms. Bacterial infections were diagnosed by means of blood cultures and analysis of sputum or bronchoalveolar lavage specimens. Routine laboratory analyses included C-reactive protein level and leukocyte count.

### PCR

Nasopharyngeal samples and bronchoalveolar lavage specimens were collected from the patients and either processed within 2 hours or refrigerated at −20°C. PCR-based detection of influenza A(H1N1)pdm09 and seasonal influenza A and B strains was performed by 1-step real-time reverse transcription PCR (*16*). Total RNA from respiratory samples was isolated with the QIAamp Viral RNA Mini Kit (QIAGEN, Hilden, Germany), according to the manufacturer’s protocol and reverse transcribed by using random hexamers from the Transcriptor First Strand cDNA Synthesis Kit (Roche, Mannheim, Germany). Reverse transcription PCR analysis was performed with 5.0 μL RNA by using the RNA Virus Master Kit (Roche,) and the LightCycler 480 System (Roche) under the following conditions: 8 min at 50°C; 30 s at 95°C; 50 cycles of 5 s at 95°C, 20 s at 60°C and 1 s at 72°C. The primer pairs were used at a concentration of 10 μM.

### Statistical Analysis

To describe the temporal distribution of admitted influenza cases, we aggregated cases by calendar week and compared our data with data from the German surveillance system of mandatory notifiable, laboratory-confirmed influenza cases (*17*). We summarized demographic and clinical data for time and severity. Frequencies were compared by using χ^2^ test or Fisher exact test for categorical variables and the Student *t* test or Mann-Whitney U-test for continuous variables, as appropriate. Identified risk factors with a p value <0.2 in the univariate analysis were included in a multivariate logistic regression model to assess independent association with severity. In a stepwise backward procedure, exposures with p>0.05 were excluded from the model. All comparisons with p<0.05 were considered statistically significant. We used Stata version 11 SE (StataCorp. LP, College Station, TX, USA) for all statistical analyses.

## Results

### Descriptive Epidemiology

We identified 178 hospitalized patients that fulfilled our case definition and included them in the study group. In the 2009 influenza pandemic season, August 2009 through April 2010, a total of 102 patients with influenza A(H1N1)pdm09 infection were admitted to the Heidelberg University Hospital. In the first postpandemic season (December 2010–March 2011), the number decreased to 76 patients. During the pandemic season, no influenza cases other than influenza A(H1N1)pdm09 infection were observed, whereas in the first postpandemic season, 4 patients received a diagnosis of influenza B virus infection. However, we did not include these influenza B–infected patients in this study.

### Epidemic Curve and Age Distribution of Influenza A (H1N1) Case-Patients

**Figure 1:**
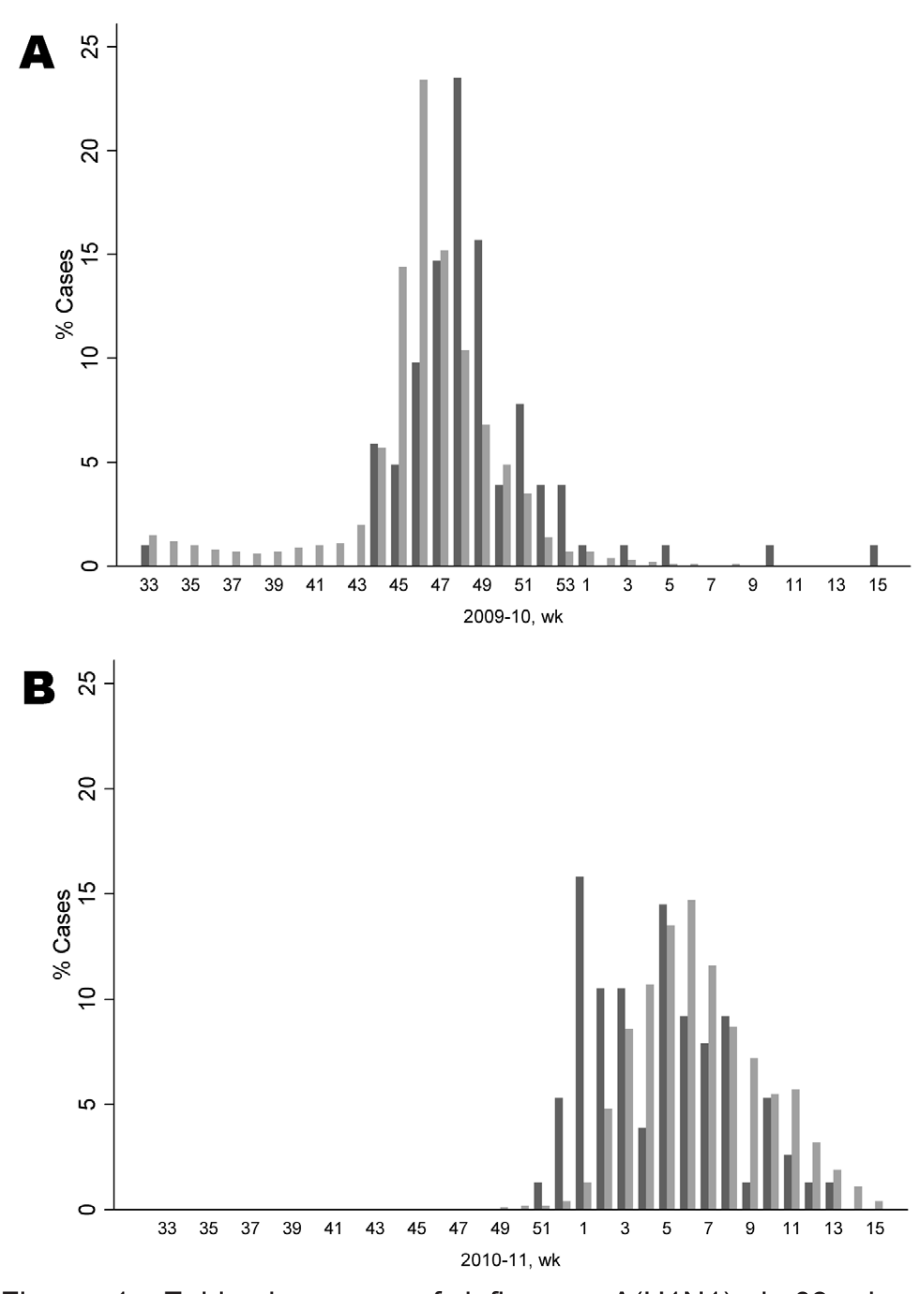
Epidemic curve of influenza A(H1N1)pdm09 virus infections. A) Season 2009–10 and B) season 2010–11. Weeks are indicated; black indicates influenza A(H1N1)pdm09 cases found in the study group at University Hospital Heidelberg; gray indicates influenza cases in Germany.

We charted the temporal distribution of influenza case-patients admitted to the University Hospital Heidelberg by date of first positive laboratory result, compared with influenza cases notified to the German national surveillance system by date of notification (Figure 1). The first observed admission occurred in August 2009 when only sporadic cases of influenza were observed in Germany. The number of admissions increased in October during the first wave with measurable effects of disease at the population level, preceding the usual beginning of the seasonal epidemic by 3 months. The distribution patterns of admissions to Heidelberg University Hospital and notified cases in Germany are similar, both in the pandemic and postpandemic year. In the 2010–11 season, the first cases of influenza were diagnosed in late December in Heidelberg, which is consistent with the usual start of the influenza season.

**Figure 2:**
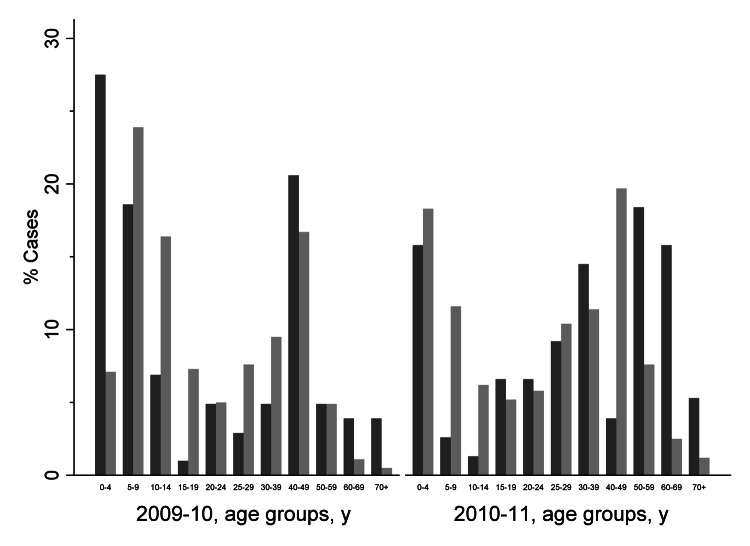
Age distribution of persons with influenza A(H1N1)pdm09 virus infections, winter 2009–10 and winter 2010–11. Black indicates influenza A(H1N1)pdm09 cases found in the study group at University Hospital Heidelberg; gray indicates influenza cases in Germany.

The distribution of influenza cases among age groups is shown in Figure 2. In the pandemic season >50% of cases from the German national surveillance system were in adolescents (5–19 years of age). In the subsequent season, this pattern shifted to younger children with >50% of cases in patients <14 years of age. The age distribution of patients admitted to the University Hospital of Heidelberg differed from the German national data with higher rates of children <10 years of age in 2009–10 and higher rates in persons >50 years of age in the 2010–11 season.

### Comparison of the 2 Seasons

The demographic and clinical characteristics of patients by influenza season are compared in Table 1. In both seasons, the sex distribution was balanced (55% male patients in 2009–10 vs. 47% in 2010-11). However, patients in the first postpandemic season tended to be older (mean age 38 vs. 18 years, p<0.05) than patients in the pandemic season and had a higher proportion of concomitant medical conditions (75% vs. 51%, p<0.05). Furthermore, an increased severity was observed in 2010–11 with a doubled mean duration of hospitalization (13.9 days vs. 7.6 days, p<0.05) and a tripled rate of patients with severe disease (46% vs. 14%, p<0.05). The mortality rate was 12% in 2010–11 compared with 5% in 2009–10 (p = 0.05).

**Table 1:** Demographic and clinical characteristics of influenza infected patients stratified by season, 2009–10 and 2010–11, University Hospital Heidelberg, Germany*

Characteristic	No. (%) patients		p value
	2009–10	2009–10	
Total no. admitted patients	102	76	
Male sex	56 (55)	36 (47)	0.32
Age, mean	18.2	38	<0.05
Days in hospital	7.6	13.9	<0.05
Admitted to ICU	14 (14)	35 (46)	<0.05
Severely diseased	15 (15)	35 (46)	<0.05
Died	5 (5)	9 (12)	0.05
Pregnant	4 (4)	5 (7)	0.50
Pneumonia	29 (28)	33 (43)	<0.05
Mechanical ventilation	11 (11)	24 (32)	<0.05
Underlying medical condition	52 (51)	57 (75)	<0.05
Immunosuppression	22 (22)	41 (54)	<0.05
Cancer	2 (2)	4 (5)	0.23
Blood malignancy	10 (10)	15 (20)	0.06
Solid organ transplant	7 (7)	14 (18)	<0.05
Autoimmune	5 (5)	6 (8)	0.41
Other	2 (2)	3 (4)	0.65
Chronic lung disease	9 (9)	13 (17)	0.10
Cardiovascular disease	25 (25)	33 (43)	<0.05
Renal impairment	9 (9)	20 (26)	<0.05
Diabetes	6 (6)	11 (14)	0.05
Metabolic dysfunction	15 (15)	13 (17)	0.66
Neurologic impairment	15 (15)	8 (11)	0.41
CRP level on admission, mg/L†	34.7 (range <2.0–213.6)	69.2 (range <2.0–381.4)	<0.05
Leukocyte count on admission, /nL†	9.9 [range 0.1–108.7)	9.1 (range 0.9–48.8)	

*ICU, intensive care unit; CRP; C-reactive protein. †Or the following day if not tested on admission.

### Comparison of Patients with Severe and Nonsevere Disease

Of the 178 patients with influenza A(H1N1)pdm09 infection hospitalized in both seasons, 50 patients (28.1%) had a severe course of infection; of these, 14 died. In univariate analysis, severity of disease was significantly associated with age, presence of pneumonia as well as with a history of an underlying medical condition, in particular, immunosuppression, chronic lung disease, cardiovascular disease, renal impairment, and diabetes (Table 2). In multivariate analysis, 4 independent risk factors for a severe clinical outcome were identified (Table 3): age, male sex, chronic lung disease, and infection in the postpandemic season were significantly associated with severity of disease. The rate of bacterial co-infection increased from 10% to ≈30% in the postpandemic season, but this rate may have been underestimated because microbiologic investigations were not performed routinely for all patients (data not shown).

**Table 2:** Demographic and clinical characteristics of influenza- infected patients stratified by severity, University Hospital Heidelberg, 2009–10 and 2010–11, Germany*

Characteristic	No. (%) patients		p value
	Nonsevere diseases	Severe disease	
Total no. admitted patients	128	50	
Male sex	61 (48)	31 (62)	0.08
Age, y, mean	18.4	47.9	<0.05
Days in hospital	6.4	20.2	<0.05
Pregnant	8 (6)	1 (2)	0.45
Pneumonia	22 (17)	40 (80)	<0.05
Underlying medical condition	64 (50)	45 (90)	<0.05
Immunosuppression	35 (27)	28 (56)	<0.05
Cancer	1 (1)	5 (10)	<0.05
Blood malignancy	12 (9)	13 (26)	<0.05
Solid organ transplant	16 (13)	5 (10)	0.64
Autoimmune	6 (5)	5 (10)	0.19
Other	2 (2)	3 (6)	0.11
Chronic lung disease	11 (9)	11 (22)	<0.05
Cardio vascular disease	29 (23)	29 (58)	<0.05
Renal impairment	14 (11)	15 (30)	<0.05
Diabetes	5 (4)	12 (24)	<0.05
Metabolic dysfunction	16 (13)	12 (24)	0.06
Neurologic impairment	16 (13)	7 (14)	0.79
CRP level on admission, mg/L†	34.6 (range 1.99–213.6)	86.7 (range 1.99–381.4)	<0.05
Leukocyte count on admission, /nL†	9.4 (range 1.9–108.7)	10.1 (range 0.1–48.8)	0.71

*CRP, C-reactive protein. †Or the following day if not tested on admission.

**Table 3:** Factors independently associated with severe clinical outcome, University Hospital Heidelberg, 2009/10–2010/11, Germany*†

Characteristic	No. patients with severe disease	Total no. patients	Relative risk (95% CI)	p value
Age, y				
0–14	5	85	1 (Ref)	
15–64	33	76	6.61 (2.83-15.42)	<0.01
>65	12	17	9.55 (3.94-23.13)	<0.01
Season				
2009–10	15	102	1 (Ref)	
2010–11	35	76	1.64 (1.02-2.62)	0.04
Chronic lung disease				
No	39	156	1 (Ref)	
Yes	11	22	1.89 (1.16-3.09)	0.01
Sex				
F (not pregnant)	18	77	1 (Ref)	
F (pregnant)	1	9	0.36 (0.06-2.36)	0.29
M	31	92	1.67 (1.09-2.57)	0.02

*Ref, reference category for risk calculations. †Variables (listed in Table 1) that did not reach the significance level of 5% are omitted.

Demographic and clinical characteristics of influenza patients with a fatal outcome are shown in Table 4. Notably, all 14 case-patients who died had an underlying medical condition; 4 of 14 had multiple myeloma.

**Table 4:** Demographic and clinical characteristics of patients infected with influenza A(H1N1)pdm09 who died, Germany, 2010–11*

Patient no.	Age, y/sex	Underlying condition	Bacterial coinfection	Pneumonia type (per chest radiograph)	Complications (other than pneumonia)	Antiviral therapy	Mechanical ventilation	Length of hospital stay, d
1	28/M	Lliver/kidney transplant	No	Bipulmonary	Renal and liver failure, SIRS	Yes	Yes	13
2	75/F	CHD, COPD, post breast cancer	No	Unilobar	Myocardial infarction	Yes	Yes	37
3	68/M	Multiple myeloma	No	Bipulmonary	Pneumothorax	Yes	Yes, + ECMO	56
4	71/M	Multiple myeloma	No	Bipulmonary		Yes	Yes, + ECMO	19
5	18/F	None	No	No	Myocarditis	No	Yes	2
6	57/F	Multiple myeloma	*Enterococcus faecium*	Bipulmonary	RSV coinfection	Yes	Yes, + NO	12
7	52/F	Multiple myeloma	No	Bipulmonary		Yes	Yes, + NO	17
8	85/F	Parkinson disease, epilepsy	No	Bipulmonary	Myocardial infarction	No	Yes, + NO	4
9	65/F	Esophageal carcinoma	No	Bipulmonary	ARDS	Yes	Yes, + NO, + ECMO	52
10	61/F	PBC, chronic hepatitis B, diabetes, COPD	*Enterococcus cloacae, Achromobacter* sp.	Bipulmonary	Renal and liver failure, sepsis, lung edema	Yes	Yes, + NO	53
11	45/F	Acute leptospirosis	*E. faecium, Leptospira* sp.	Bipulmonary	Renal and liver failure	Yes	Yes	13
12	49/M	Colitis ulcerosa	*Escherichia coli*	Bipulmonary	Perforation of colon, hemorrhagic shock, lung edema	Yes	Yes, + NO, + ECMO	8
13	61/F	CHD, CKD	*Klebsiella pneumoniae*	Bipulmonary	Septic shock	Yes	Yes, + NO	28
14	53/F	Liver cirrhosis	*E. faecium*	Bipulmonary	Renal and liver failure, septic shock, CMV reactivation, aspergillosis	Yes	Yes	43
15	52/M	Multiple myeloma, post allogenic transplant	*E. faecium*	Bipulmonary	GvHD 4, cerebral hemorrhage, CMV reactivation, progressive myeloma	Yes	Yes	46

*SIRS, systemic inflammatory response syndrome; CHD, congestive heart disease; COPD, chronic obstructive pulmonary disease; ECMO, extracorporeal membrane oxygenation; RSV, respiratory syncytial virus; NO, nitric oxide; ARDS, acute respiratory distress syndrome; PBC, primary biliary cirrhosis; CKD, chronic kidney disease; CMV, cytomegalovirus; GvHD, graft versus host disease.

### Multivariable Analysis of Risk Factors for Disease Severity

Our multivariable logistic regression model identified 4 independent risk factors for severe clinical disease (Table 3). Older, male patients with any chronic lung disease were more affected in the postpandemic season. The other variables mentioned in Table 2 did not reach statistical significance and were therefore not included in our final model.

## Discussion

In our study, the rate of severely diseased patients with influenza A(H1N1)pdm09 virus infection increased 3-fold in the first postpandemic season, resulting in an in-hospital mortality rate of 12% compared with 5% in the pandemic season. Also, the length of hospital stay doubled in the postpandemic season, and the need for mechanical ventilation and ICU admission were significantly higher. We identified older age, male sex, any chronic lung disease, and diagnosis in the postpandemic season as independent risk factors for a severe clinical course of disease. Our study was performed in a retrospective manner without standardized data collection. Information in the medical records can be incomplete, especially for the classification for severe outcome and underlying medical conditions. Therefore, no established scoring system like the pneumonia severity index could be used in classifying severe clinical course.

Influenza A(H1N1)pdm09 virus in the 2009–10 season affected mainly children and young adults in Germany (*18*). Most hospitalized case-patients in our study group in 2009–10 were young patients, confirming that children and young adults were the age category mostly affected by the pandemic. The low prevalence of older patients in the pandemic season has been attributed to residual immunity against influenza A(H1N1)pdm09 strains in persons born before 1950 (*19*). Previous studies found that current (*20*,*21*) and past pandemics (*22*) affected mainly younger patients. This finding is in contrast with findings of epidemiologic features of seasonal influenza, for which hospitalizations are more common among persons ≥65 years of age and those <15 years.

The highest increase in seroprevalence between pre- and postpandemic periods was observed among patients 18–29 years of age in Germany (*23*). In the United Kingdom, population-based evaluation of serologic immunity to the pandemic strain after the pandemic season suggested that susceptibility was lowest in age groups <15 years, with substantial remaining susceptibility in the those 15–44 years (*24*). Because the remaining susceptibility in children is limited, the probability of extensive illness in this age group with influenza A(H1N1)pdm09 virus in the 2010–11 season was unlikely in the absence of substantial antigenic change in the pandemic virus. Early in the 2010–11 season, high rates of positivity are most marked in those ages 15 to 44 years, the major group contributing to hospital admissions and death (*25*). This result is in contrast to findings in the 2009–10 pandemic when highest rates of infection in the community were observed in children. The increase in severe courses of infection and requirement for critical care in 2010–11 might reflect the effects of influenza A(H1N1)pdm09 illness in the remaining susceptible adults and risk groups in the population, primarily older patients with coexisting conditions—the historically recognized risk factors for seasonal influenza.

Younger age, chronic coexisting conditions, morbid obesity, and bacterial coinfection have been reported as independent risk factors for severe disease in the pandemic season (*3*,*4*). Of hospitalized patients with severe influenza A(H1N1)pdm09 infection, 15%–20% were immunosuppressed in the pandemic season (*3*,*4*). These data are in line with a rate of 27% of immunosuppressed patients in our cohort. It might be speculated that hematologic malignancy or treatment of hematologic disorders is an independent risk factor for fatal disease. Studies involving patients with pandemic influenza A(H1N1)pdm09 infection found that although older patients had the lowest estimated incidence rate, they also had the highest case-fatality rate (*6*). Delayed hospital admission and delayed antiviral therapy have been associated with an unfavorable outcome in the general population and among solid-organ recipients (*12*). The case-fatality rate increased from 5% to 13% in patients we studied; a lower rate of 3% in the pandemic season had been reported previously (*26*). Bacterial coinfection is common in case-patients with a fatal outcome (*27*–*29*) and has also been observed in our patients who died. Thus, timely administration of antiviral and/or antibacterial drug therapy is indicated in high-risk patients.

The increase of severe and even fatal cases in hospitalized patients with influenza A(H1N1)pdm09 infection in the subsequent season of the pandemic has also been reported by the Novel Influenza A (H1N1) Study Group of the Spanish Network for Research in Infectious Diseases (*30*) in Spain. Severe clinical courses have been described for patients with underlying conditions, pregnant women, and obese patients (*4*,*10*). In 2009, this study group observed similar rates of severe disease (*31*) to our observations, namely, 28% with pneumonia and 15% with severe disease. Molecular characterization of influenza A(H1N1)pdm09 viruses circulating in 2010 revealed only a minor genetic drift compared with early isolates from 2009 (*25*), which most likely cannot be attributed for the change in clinical outcome.

In conclusion, we found notable epidemiologic changes and an increased severity of influenza A(H1N1)pdm09 infections in the first postpandemic influenza season. These findings reinforce the need to identify and protect groups at highest risk for adverse outcomes.
